# Family History of Autoimmune Disease in Patients with Aicardi-Goutières Syndrome

**DOI:** 10.1155/2012/206730

**Published:** 2012-11-11

**Authors:** Johanna L. Schmidt, Ivana Olivieri, Jodie M. Vento, Elisa Fazzi, Heather Gordish-Dressman, Simona Orcesi, Adeline Vanderver

**Affiliations:** ^1^Department of Neurology, Children's National Medical Center, Washington, DC 20010, USA; ^2^Center for Genetic Medicine Research, Children's National Medical Center, Washington, DC 20010, USA; ^3^Unit of Child Neurology and Psychiatry, C. Mondino National Institute of Neurology Foundation, Pavia 27100, Italy; ^4^Department of Child and Adolescent Neuropsychiatry, University of Brescia, Brescia 25123, Italy

## Abstract

*Purpose*. The purpose of this study was to explore anecdotal evidence for an increase in the prevalence of autoimmune diseases in family members of patients with Aicardi-Goutières syndrome (AGS). *Methods*. Pedigrees of patients and controls were analyzed using chi-square and logistic regression to assess differences in reports of autoimmune disease among family members of cases and controls. Data was collected at Children's National Medical Center in Washington, DC, USA and at the International Aicardi-Goutières Syndrome Association Scientific Headquarters, C. Mondino National Institute of Neurology in Pavia, Italy. *Results*. The number of individuals with reported autoimmune disease is significantly related to having a family member with AGS (*χ* ^2^ = 6.25, *P* = 0.01); 10% (35/320) of relatives of patients with AGS had a reported autoimmune disease diagnosis compared to 5% (18/344) of relatives of controls. There was a greater percent of maternal relatives of patients with AGS reporting autoimmune disease (14.6%), compared to controls (6.8%), with the association being statistically significant. The association was not significant for paternal relatives. *Conclusion*. The prevalence of autoimmune disease in relatives of children with AGS is significantly increased compared to controls. More research is needed to better understand this association.

## 1. Introduction

Aicardi-Goutières syndrome (AGS) is a heritable neurologic disorder with an immune basis. Patients most typically present early in life with increased cerebrospinal fluid (CSF) interferon alpha and markers of inflammation, elevated liver enzymes, thrombocytopenia, intracranial calcifications and leukoencephalopathy. Patients with AGS usually demonstrate severe neurologic dysfunction and life-long disability. 

The immune basis of AGS was originally suspected by Aicardi and Goutières, as a persistent CSF pleocytosis was seen in affected infants. Infants presented with what appeared to be a congenital infection [1], and reports of elevations of CSF IFN*α* [2] and neopterin [3, 4] further suggested that an immune process was at play. However, it was not until the identification of AGS related mutations in nucleases, including TREX1 and the three constituent subunits of RNase H2, as well as a nonnuclease, SAMHD1, that the relationship between innate cellular immunity and AGS began to be more fully understood. 

Discovery of the genes associated with AGS allowed for further definition of the phenotype. Most patients with AGS were found to have homozygous or compound heterozygous changes in these genes. AGS was also found to be allelic with Cree encephalitis and an inherited infantile systemic lupus erythematosus [5–8]. Heterozygous mutations in AGS related genes have also been found in rare patients with systemic lupus erythematosus (SLE) [9, 10] and familial chilblain lupus (FCL) [11–13], which are both autoimmune disorders. Studies in large populations of SLE patients suggest that* TREX1* single nucleotide polymorphisms may also be related to neurologic manifestations and the presence of autoantibodies [10]. Additionally, mutations in *TREX1* have been reported in patients with retinal vasculopathy with cerebral leukodystrophy (RVCL) [14].

Of the five genes currently known to be associated with AGS, four, *TREX1* and *RNASEH2A-C*, encode proteins that have nucleic acid metabolizing functions, or nucleases. The fifth gene *SAMHD1*, encodes a protein that while not specifically a nuclease is thought to degrade nucleic acid precursors [15] and is upregulated by immune-stimulatory DNA [16]. Mutations in these genes are thought to result in the accumulation of endogenous nucleic acids. Growing evidence suggests that this accumulation provokes a type 1 interferon response that results in the development of pathogenic cytokines and autoantibodies that target the brain and other organs [17]. 

Anecdotal observation suggests an increase in the prevalence of various autoimmune diseases in the family history of children diagnosed with AGS. However, despite the evidence of AGS related mutations in some individuals from SLE populations, this has never been explored in families of AGS affected persons. The purpose of this study was to investigate this anecdotal observation to determine if there is an increase in prevalence of autoimmune disease in families who have children with a diagnosis of AGS, as compared to control families. 

## 2. Materials and Methods

To investigate the possible increase in prevalence of autoimmune disease in family members of children with AGS, pedigrees were analyzed from families with AGS and controls who either had a definitive diagnosis other than AGS or who had unsolved (i.e., undiagnosed) leukodystrophies without any features of AGS (including clinical and neuroradiological findings). AGS cases were defined by having consistent clinical features (elevated alpha interferon and white blood cells in CSF, central nervous system calcifications, and leukoencephalopathy and no other identifiable etiology) and by confirmed mutations in any of the known AGS related genes. 

Autoimmune disease is a broad term and, for the purposes of this study, was considered to include autoimmune thyroiditis leading to hypothyroidism, and other autoimmune thyroid dysfunction, inflammatory bowel disease, type I diabetes, rheumatoid arthritis, multiple sclerosis, Raynaud syndrome, psoriasis, scleroderma, Addison disease, and Kawasaki disease, [18–20] as well as the above mentioned autoimmune diseases (i.e., systemic lupus erythematosus and familial chilblain lupus) already known to be associated with AGS related genes.

The presence of autoimmune disease was investigated in case and control families using semistructured interviews to inquire about autoimmune disease generally and about the presence of specific autoimmune diseases (lupus, thyroid dysfunction, inflammatory bowel disease, type I diabetes, rheumatoid arthritis, multiple sclerosis, and autoimmune skin disorders). Data was collected between September 2008 and October 2011. Pedigrees for AGS subjects were drawn during AGS family clinic/conferences in 2008 and 2011 at Children's National Medical Center, or during clinical encounters in Washington DC or in Pavia, Italy. Pedigrees for control subjects were drawn during clinical encounters in Washington, DC by the same staff as performed AGS pedigrees. Pedigrees were excluded if there was insufficient information from one side of the family or if the patient was adopted and family history information was limited. Consanguineous families were included in both cases and controls. Pedigrees were drawn by genetic counselors or a pediatric neurologist with special expertise in biochemical genetics and leukodystrophy. 

Data collection was part of a larger data collection effort within the European NIMBL (Nuclease Immune Mediated Brain and Lupus-like conditions) project. Italian families who participated to this study were enrolled in NIMBL in collaboration with the International Aicardi Goutieres Syndrome Association (IAGSA). United States families were enrolled in NIMBL in collaboration with the Myelin Disorders Bioregistry Project. The project was approved by the Institutional Review Board at Children's National Medical Center and the ethical review boards at the Child Neurology and Psychiatry Unit at the C. Mondino National Institute of Neurology, Pavia, Italy. 

The prevalence of autoimmune disease was investigated all relatives combined, in parents only, and in second degree relatives only. In this study, half-siblings of parents were also considered to be second-degree relatives and were included in the analysis. Siblings and half-siblings of patients were not included in the analysis, as most of them were too young to have exhibited any features of autoimmune disease and may or may not develop autoimmune disease in the future.

Chi-square and/or logistic regression were used to assess differences between overall relatives, between maternal relatives and paternal relatives, between mothers and fathers, and between second-degree relatives in cases and controls. 

## 3. Results

Pedigrees of families of children with AGS (*N* = 31) were compared with control pedigrees of families of children without AGS (*N* = 31). The US site collected family history information on 17 of the AGS families, while the Italian group collected family history on 14 families. Pedigrees were collected in a prospective fashion. 

Controls were all from the Myelin Disorders Bioregistry at Children's National Medical Center in Washington, DC. Controls were patients who either had a clinical or molecular diagnosis other than AGS, or had an undetermined diagnosis but without features of AGS. Control diagnoses included Alexander disease, metachromatic leukodystrophy (MLD), hypomyelination with hypogonadotrophic hypogonadism and hypodontia (4H) syndrome, mucopolysaccharidosis IIIc, multiple sulfatase deficiency, congenital cytomegalovirus (CMV), acute disseminated encephalomyelitis (ADEM), and peroxisomal disorders. 

Comparisons of autoimmune disease diagnoses were assessed in relatives of AGS patients and control patients using chi-square and logistic regression (Tables 1 and 2). First, a chi-square test was performed to assess differences in reports of autoimmune diseases in the two groups. The number of relatives with reported autoimmune disease is significantly related to having a family member with AGS (*χ* ^2^ = 6.25, *P* = 0.01): there were 320 total relatives of patients with AGS, and 35 (10.9%) had reported autoimmune disease diagnoses, compared to 5.2% (18/344) controls. Thus, the prevalence of autoimmune disease in AGS families was more than twice that of the control families. 

Next, a chi-square calculation was performed on maternal versus paternal relatives. There was a greater percent of maternal relatives with reported autoimmune disease (24/164, or 14.6%) compared to controls (12/176, or 6.8%), and this association was statistically significant (*P* = 0.03). For paternal relatives, the association between having diagnosis of an autoimmune disease and having a relative with AGS was not significant. 

Using logistic regression, when looking at all first degree relatives (mothers and fathers of cases and controls) alone, patients with AGS were not significantly more likely to have a parent with a reported autoimmune disease (OR = 2.24; *P* = 0.29; 95% CI = 0.51–9.91). Of note, however, all of the parents who were reported to have autoimmune disease were in the US-based cohort of families (Table 1). If the US cohort is considered alone (Table 3), patients with AGS were statistically significantly more likely to have a parent with reported autoimmune disease (OR = 5.09; *P* = 0.040; 95% CI= 1.07–24.02). Reports of autoimmune diseases in first degree relatives of children with AGS included diagnoses of: thyroid dysfunction, psoriasis, lupus-like symptoms, and ulcerative colitis. 

 Logistic regression was again used for second-degree relatives alone (grandparents, aunts, and uncles of patients (Table 2)). Patients with AGS were significantly more likely to have at least one second degree relative with autoimmune disease compared to controls (OR = 2.88, *P* = 0.044, 95% CI = 1.03–8.07) (Table 2). A linear trend test was performed to determine if the odds ratio increased with increasing numbers of second degree relatives with reported autoimmune disease. There was evidence suggestive of a trend in the number of maternal family members (*P* = 0.086), but not in the number of paternal family members. Reports of autoimmune disease diagnoses in this group of second-degree relatives of AGS included: chilblains, sarcoidosis, psoriasis, lupus, inflammatory bowel disease, Raynaud's, scleroderma, multiple sclerosis, thyroid dysfunction, and rheumatoid arthritis. Individual numbers were too small to make meaningful comments about subsets of autoimmune disorders, specifically SLE, seen in this population.

 Because there was a difference in the report of autoimmune disease diagnoses between females (mothers) versus males (fathers), an assessment of differences in female versus male second-degree relatives' report of autoimmune disease diagnoses was performed. Among the 29 second-degree relatives in the AGS group who were reported to have autoimmune disease diagnoses, 21 (72.4%) were female and 8 (27.6%) were male. Among the 15 individuals in the control group who were reported to have an autoimmune disease diagnosis, 10 (66.7%) were female and 5 (33.3%) were male. 

## 4. Discussion

Anecdotal evidence of an increased prevalence of autoimmune disease in family members of patients with AGS prompted this analysis of pedigrees of AGS patients in comparison to control families. AGS is part of a growing group of inherited disorders in which increased alpha interferon is thought to play a substantial role in the pathogenesis [21]. Familial increases in alpha interferon has been shown to aggregate with increased risk of established autoimmune disorders such as SLE [22] and juvenile dermatomyositis [23]. Thus, it is not surprising that there is a greater percentage of relatives of patients with AGS with reported autoimmune disease compared to controls, and that this association was statistically significant. It should be noted that this statistically significant association held for maternal relatives, but not so for paternal relatives. Patients with AGS in the US cohort alone were shown to be statistically significantly more likely to have a parent with autoimmune disease compared to controls.

To explain the difference between the US and Italian cohorts, genotype was considered, since a larger proportion of in the Italian group of AGS patients had mutations in *RNASEH2B*, but removing those from the analysis did not result in any substantial change in findings. Thus, this difference is not understood and requires additional investigation. It is likely that the sample size is simply too small to detect meaningful differences. 

Statistical analysis did show that children with AGS were significantly more likely to have at least one second degree relative with autoimmune disease as compared to controls. The finding of an increase in prevalence in females as compared to males was unexpected, although it is consistent with data showing that, in general, women are 2-3 times more likely to have autoimmune disease than men [18]. Although difficult to capture due to the variability of autoimmune diseases, the prevalence of autoimmune disease in the US may be approximately 1/31 or 3.2% [18]. Our control population was found to have a slightly higher prevalence of autoimmune diseases, but our case population was found to have significantly more than this rate. The slight increase in prevalence in controls may be due to the fact that our controls were drawn from children with other neurogenetic diseases. Assessing differences in prevalence of autoimmune diseases in pedigrees of AGS families and pedigrees of families without any neurogenetic disease may provide a more accurate comparison. 

It is unclear at this time whether the increased prevalence of autoimmune disease in relatives of AGS patients is directly related to mutation status in these relatives. Of note, although parents were generally presumed to be carriers of mutations in AGS-related genes, the genotype of second degree relatives with reported autoimmune disorders was not known. In addition, it is unknown whether other genetic modifiers exist that could change the phenotypic expression of mutations in AGS related genes, which may predispose related individuals to autoimmune diseases. Finally, other factors may exist, including the fact that families with a diagnosis of AGS may be more attentive to symptoms of autoimmune disorders than control families, or that other familial confounders may exist, such as diet, environment or infection. This may explain differences seen between the US and Italian cohorts, for example. Therefore, there is not enough evidence or understanding at this time to claim that individuals with diagnosed autoimmune diseases should be screened for mutations in AGS-related genes, or that family members of AGS patients known to carry a heterozygous mutation should be screened for autoimmune disorders. 

However, it is of note that *TREX 1* mutations have been found in patients with SLE. The relative risk for the development of SLE among those who carry *TREX1* variants has been found to be 25.3 (95% CI = 5.6–232.0) in one cohort (*N* = 317) [9]. In another recently studied large cohort of SLE patient (*N* = 8730), mutations in *TREX1* one occurred at a frequency of 0.5% [10]. This data, in addition to our finding of increased prevalence of autoimmune disease in family members of AGS patients, suggests the need for further research into genotype-phenotype correlations of AGS related mutations and autoimmune disease. 

## 5. Limitations

 Because family history data was obtained by report of parents of cases and controls, and not by primary analysis of family members' medical records, data may be inaccurate in both cases and controls. It is possible that both families of patients with AGS and control patients experienced recall bias or that they were not aware of autoimmune diagnoses in the family, and, thus, prevalence of autoimmune disease in both groups may be underreported. Additionally, because the semi-structured interviews for family history information were done at different times and by different investigators, not all cases and controls were asked about the same conditions in the same way, although every attempt was made to make the data as consistent as possible, including the use of the same group of investigators to collect control and AGS pedigrees. 

 One consanguineous case family and one consanguineous control family were included in the analysis. This was unlikely to have had an effect on the data, since second degree relatives of affected children were likely still only at 50% risk of having inherited a heterozygous mutation from a parent. There were no reports of affected individuals in either the case or control family in previous generations.

 AGS is a rare disorder, and therefore, sample sizes are small. However, the disease may be underdiagnosed. As awareness of AGS grows and additional patients are diagnosed, more family history data will be available. Also, as the understanding of the autoimmune nature of this disease improves, more robust conclusions can be drawn about the association and risk for developing autoimmune disease in obligate or presumed carriers. 

## 6. Conclusion

 In this evaluation of family pedigrees from patients with Aicardi-Goutières Syndrome and control subjects, relatives of patients with AGS reported autoimmune disease diagnoses more frequently than family members of control patients; patients with AGS in the US-based cohort alone were statistically more likely to have a first-degree relative with an autoimmune disease and the entire cohort was found to be statistically more likely compared to controls to have at least one second-degree relative with autoimmune disease. Female relatives reported autoimmune disease diagnoses more frequently than male relatives. This data, in addition to the presumed autoimmune nature of AGS and the fact that genes that cause AGS (i.e., *TREX1 *and *SAMHD1*) are allelic to those that can cause SLE and FCL, warrants further study into the association between heterozygous mutations in AGS-related genes and autoimmune disease. There is not enough data to suggest that heterozygous carriers of mutations in AGS-related genes are at risk for developing autoimmune disease, nor that those individuals with autoimmune disease should be screened for mutations in these genes, but further work is needed to better understand the association. 

## Figures and Tables

**Table 1:** History of autoimmune disease in parents of cases and controls.

Characteristic	AGS cases	Control	OR	*P* value	95% CI
Maternal history of AI (yes/no)					
No	26 (83.9%)	28 (90.3%)	1.00		
Yes	5 (16.1%)	3 (9.7%)	1.75	0.453	0.39–8.27

Paternal history of AI (yes/no)					
No	30 (96.8%)	31 (100.0%)	1.00		
Yes	1 (3.2%)	0 (0.0%)	—	0.313*	—

Maternal and/or paternal history of AI (yes/no)					
No	25 (80.7%)	28 (90.3%)	1.00		
Yes	6 (19.4%)	3 (9.7%)	2.24	0.288	0.51–9.91

*P value from chi-squared test due to zero control subjects with a paternal history of AI.

**Table 2:** History of autoimmune disease in second degree relatives of cases and controls.

Characteristic	AGS cases	Control	OR	*P* value	95% CI
All second degree relatives both matrilineal and patrilineal with history of AI (yes/no)					
No	11 (35.5%)	19 (61.3%)	1.00		
Yes	20 (64.5%)	12 (38.7%)	2.88	0.044	1.03–8.07

**Table 3:** Comparison of autoimmune disease reported in first degree relatives in US-based cohort only.

Characteristic	AGS cases	Control	OR	*P* value	95% CI
Maternal and/or paternal history of AI (yes/no)					
No	11 (64.7%)	28 (90.3%)	1.00		
Yes	6 (35.3%)	3 (9.7%)	5.09	0.040	1.07–24.02
